# Meta-genome-wide association studies identify a locus on chromosome 1 and multiple variants in the MHC region for serum C-peptide in type 1 diabetes

**DOI:** 10.1007/s00125-018-4555-9

**Published:** 2018-02-05

**Authors:** Delnaz Roshandel, Rose Gubitosi-Klug, Shelley B. Bull, Angelo J. Canty, Marcus G. Pezzolesi, George L. King, Hillary A. Keenan, Janet K. Snell-Bergeon, David M. Maahs, Ronald Klein, Barbara E. K. Klein, Trevor J. Orchard, Tina Costacou, Michael N. Weedon, DCCT/EDIC Research Group, Richard A. Oram, Andrew D. Paterson

**Affiliations:** 1 Genetics and Genome Biology Program, Peter Gilgan Centre for Research and Learning (PGCRL), The Hospital for Sick Children, 686 Bay Street, Toronto, ON M5G 1H3 Canada; 2 University Hospitals Case Western Medical Center, Cleveland, OH USA; 3 Lunenfeld–Tanenbaum Research Institute, Sinai Health System, Toronto, ON Canada; 4 Dalla Lana School of Public Health, University of Toronto, Toronto, ON Canada; 5 Department of Mathematics and Statistics, McMaster University, Hamilton, ON Canada; 6 Division of Nephrology and Hypertension, Diabetes and Metabolism Center, University of Utah, Salt Lake City, UT USA; 7 Research Division, Joslin Diabetes Center, Boston, MA USA; 8 Department of Medicine, Harvard Medical School, Boston, MA USA; 9 Barbara Davis Center for Diabetes, University of Colorado Anschutz Medical Campus, Aurora, CO USA; 10 Department of Paediatrics, Stanford School of Medicine, Stanford, CA USA; 11 Department of Ophthalmology and Visual Sciences, University of Wisconsin, Madison, WI USA; 12 Department of Epidemiology, Graduate School of Public Health, University of Pittsburgh, Pittsburgh, PA USA; 13 Institute for Biomedical and Clinical Science, University of Exeter Medical School, Exeter, UK; 14 National Institute for Health Research, Exeter Clinical Research Facility, Exeter, UK

**Keywords:** C-peptide, Genome-wide association study, Insulin-secreting cells, Single nucleotide polymorphism, Type 1 diabetes

## Abstract

**Aims/hypothesis:**

The aim of this study was to identify genetic variants associated with beta cell function in type 1 diabetes, as measured by serum C-peptide levels, through meta-genome-wide association studies (meta-GWAS).

**Methods:**

We performed a meta-GWAS to combine the results from five studies in type 1 diabetes with cross-sectionally measured stimulated, fasting or random C-peptide levels, including 3479 European participants. The *p* values across studies were combined, taking into account sample size and direction of effect. We also performed separate meta-GWAS for stimulated (*n* = 1303), fasting (*n* = 2019) and random (*n* = 1497) C-peptide levels.

**Results:**

In the meta-GWAS for stimulated/fasting/random C-peptide levels, a SNP on chromosome 1, rs559047 (Chr1:238753916, T>A, minor allele frequency [MAF] 0.24–0.26), was associated with C-peptide (*p* = 4.13 × 10^−8^), meeting the genome-wide significance threshold (*p* < 5 × 10^−8^). In the same meta-GWAS, a locus in the MHC region (rs9260151) was close to the genome-wide significance threshold (Chr6:29911030, C>T, MAF 0.07–0.10, *p* = 8.43 × 10^−8^). In the stimulated C-peptide meta-GWAS, rs61211515 (Chr6:30100975, T/–, MAF 0.17–0.19) in the MHC region was associated with stimulated C-peptide (β [SE] = − 0.39 [0.07], *p* = 9.72 × 10^−8^). rs61211515 was also associated with the rate of stimulated C-peptide decline over time in a subset of individuals (*n* = 258) with annual repeated measures for up to 6 years (*p* = 0.02). In the meta-GWAS of random C-peptide, another MHC region, SNP rs3135002 (Chr6:32668439, C>A, MAF 0.02–0.06), was associated with C-peptide (*p* = 3.49 × 10^−8^). Conditional analyses suggested that the three identified variants in the MHC region were independent of each other. rs9260151 and rs3135002 have been associated with type 1 diabetes, whereas rs559047 and rs61211515 have not been associated with a risk of developing type 1 diabetes.

**Conclusions/interpretation:**

We identified a locus on chromosome 1 and multiple variants in the MHC region, at least some of which were distinct from type 1 diabetes risk loci, that were associated with C-peptide, suggesting partly non-overlapping mechanisms for the development and progression of type 1 diabetes. These associations need to be validated in independent populations. Further investigations could provide insights into mechanisms of beta cell loss and opportunities to preserve beta cell function.

**Electronic supplementary material:**

The online version of this article (10.1007/s00125-018-4555-9) contains peer-reviewed but unedited supplementary material, which is available to authorised users.


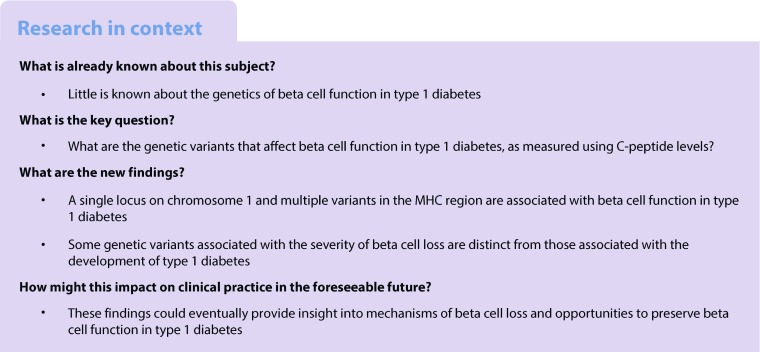


## Introduction

Type 1 diabetes develops when pancreatic beta cells fail to produce sufficient insulin to maintain euglycaemia [1]. C-peptide measurement after a standard meal (stimulated C-peptide) is the primary way to evaluate insulin secretion in type 1 diabetes [2, 3]. However, fasting/random C-peptide measurements are less demanding and feasible to obtain in large population samples. The alpha and beta chains of the proinsulin molecule are joined by C-peptide, which is removed during insulin secretion. Therefore, C-peptide is co-secreted with insulin in an equimolar ratio. Unlike insulin, C-peptide undergoes little first-pass clearance by the liver, has a much longer half-life and is not interfered with by exogenous insulin [2, 3].

Type 1 diabetes is a heterogeneous disease that can occur at any age. C-peptide levels vary at diagnosis. Early-onset type 1 diabetes (age <2–5 years) is associated with rapid beta cell loss, the presence of high-risk MHC type 1 diabetes genetic susceptibility and high titres of autoantibodies compared with late-onset disease [4]. A large proportion of people with type 1 diabetes have detectable C-peptide years after diagnosis, indicating that some beta cells are still functional [2, 5–8]. Sensitive assays have detected stimulated C-peptide in 73–100% of people with type 1 diabetes for ≥30 years [7, 8].

C-peptide preservation has been reported to be associated with favourable metabolic and clinical outcomes in type 1 diabetes. Individuals with preserved C-peptide levels maintain better long-term glycaemic control despite a lower exogenous insulin dose per body weight [3]. They are also at lower risk for both hypoglycaemia and diabetic complications, which are major health problems [3, 9–12]. The association of C-peptide with long-term diabetic complications (i.e. retinopathy and nephropathy) remains significant after adjusting for long-term glycaemic control, suggesting that at least part of the effect of C-peptide on diabetic complications is independent of its effect on glycaemic control (or, alternatively, that frequency of blood glucose/HbA_1c_ measurements is suboptimal) [3, 9]. However, association of C-peptide levels with long-term diabetic complications has not been observed in other studies [13].

There have been no family/twin studies of C-peptide levels in type 1 diabetes. In the Diabetes Control and Complications Trial (DCCT), investigations of detectable stimulated C-peptide levels in participants with type 1 diabetes (*n* = 98) and their first-degree affected relatives (*n* = 109) could not reach conclusions because of an insufficient sample size (see electronic supplementary material [ESM] Tables 1, 2). To date, more than 50 type 1 diabetes risk loci have been identified. The majority are thought to be involved in the immune system, and many are shared with other autoimmune diseases [14]. However, there have been no genome-wide association studies (GWAS) of C-peptide in type 1 diabetes (www.ebi.ac.uk/gwas, accessed 1 July, 2017) and candidate gene-association studies have investigated a limited number of genes in small populations with no replication in independent studies [15]. It is clear from other diseases that factors for the risk of a disease and its progression can be independent [16]. Here, we aimed to identify genetic loci associated with residual beta cell function in type 1 diabetes as measured by C-peptide through meta-analysis of GWAS.

## Methods

### Study design

**Fig. 1:**
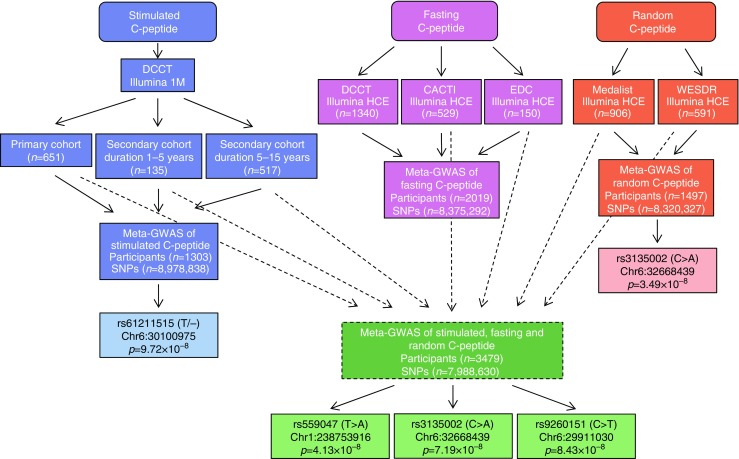
Overview of the C-peptide cross-sectional data analysis and the main findings. The dashed arrows show the studies included in the meta-GWAS of stimulated, fasting and random C-peptide. HCE, human core exome

We performed meta-GWAS of stimulated, fasting and random (non-fasting) C-peptide separately in European (confirmed by population structure analysis) participants from the following five type 1 diabetes studies (Fig. 1):

- Stimulated C-peptide: the DCCT, including: (1) primary cohort; (2) secondary cohort with diabetes duration 1–5 years; and (3) secondary cohort with diabetes duration 5–15 years
- Fasting C-peptide: (1) the DCCT (all); (2) the Coronary Artery Calcification in Type 1 Diabetes (CACTI) study; and (3) the Pittsburgh Epidemiology of Diabetes Complications (EDC) study
- Random C-peptide: (1) the Joslin 50-Year Medalist study; and (2) the Wisconsin Epidemiologic Study of Diabetic Retinopathy (WESDR)

In the analysis of stimulated C-peptide, DCCT participants were divided into three groups (a primary cohort and a secondary cohort with diabetes duration of 1–5 and 5–15 years), since the range of stimulated C-peptide levels differed among the three groups based on the inclusion/exclusion criteria. In contrast, because all DCCT participants had fasting C-peptide levels of ≤0.2 pmol/ml, the fasting C-peptide analysis was performed with all participants as a single group (‘all’).

Another meta-analysis was performed to combine the GWAS results for stimulated (DCCT), fasting (CACTI and EDC) and random (Joslin 50-Year Medalist and WESDR) C-peptide (Fig. 1). We specifically examined the associations of known type 1 diabetes loci and type 1 diabetes genetic risk scores (GRS) with C-peptide levels [14, 17]. The associations of the identified loci and type 1 diabetes loci/GRS were tested with stimulated C-peptide change over time in a subset of DCCT participants with repeated annual measures for up to 6 years (*n* = 258).

### Studies

#### DCCT

Individuals aged 13–39 years with type 1 diabetes and fasting serum C-peptide levels ≤0.2 pmol/ml were recruited between 1983 and 1989. The primary cohort included participants with 1–5 years of diabetes, no pre-existing retinopathy, normal albuminuria (<40 mg/24 h) and stimulated C-peptide ≤0.5 pmol/ml. The secondary cohort included participants with 1–15 years of diabetes, pre-existing mild retinopathy, albumin excretion rate <200 mg/24 h and stimulated C-peptide ≤0.5 and 0.2 pmol/ml for participants with diabetes duration 1–5 and 5–15 years, respectively [ESM Table 3]. Participants were randomly assigned to receive intensive or conventional insulin treatment and were monitored until 1993 [18]. C-peptide levels were measured annually for up to 6 years in those with stimulated C-peptide >0.2 pmol/ml at eligibility testing [19, 20].

#### CACTI

CACTI was an observational, population-based study of individuals with type 1 diabetes of duration >10 years at enrolment. Participants were recruited from Denver, CO, USA, between March 2000 and April 2002 [21]. Fasting serum C-peptide was measured during 2014–2016 [22] ESM Table 3].

#### EDC

This study recruited a cohort of individuals with incident, childhood-onset (<17 years of age) type 1 diabetes who were diagnosed or seen within 1 year of diagnosis (1950–1980) at the Children’s Hospital of Pittsburgh. The first participant assessments occurred in 1986–1988 [23]. Serum C-peptide was measured at follow-up year 25 [ESM Table 3]. Participants were advised to fast for ≥8 h, but 17% (*n* = 26) did not. Of the participants who failed to fast, only 15% (*n* = 4) had detectable C-peptide (unpublished data, T. J. Orchard and T. Costacou).

#### Joslin 50-year Medalist study

This was a cross-sectional study. All participants provided their original medical record or three forms of documentation of ≥50 years of insulin-dependent diabetes and also resided in the USA at the time of participation [24]. Random serum C-peptide levels were measured and validated at the Northwest Lipid Research Laboratory at the University of Washington [ESM Table 3] [13].

#### WESDR

People with diabetes who received primary care in an 11-county area in southern Wisconsin from 1979 to 1980 were recruited and subsequently followed for diabetic complications [25, 26]. The participants were all younger-onset individuals who were diagnosed before 32 years of age and treated with insulin. Random serum C-peptide levels were measured at years 4 and 10 of follow-up [ESM Table 3] [27], with the most recent results used for analysis.

### Genotyping and imputation

Genotyping was performed using Illumina 1M [28] and HumanCoreExome BeadArrays (Illumina, San Diego, CA, USA) in the DCCT. Illumina HumanCoreExome BeadArrays were used for genotyping in the CACTI, EDC, Joslin 50-Year Medalist and WESDR studies. Ungenotyped autosomal SNPs were imputed using 1000 Genomes data (phase 3, v5) (see ESM Methods for more details) [29].

Genotype dosage data from Illumina 1M BeadArrays were used to analyse stimulated C-peptide in the DCCT, as approximately 3 million more SNPs were imputed with good quality (INFO ≥0.8); and genotype dosage data from HumanCoreExome BeadArrays were used for the remaining analyses in all studies. SNPs with minor allele frequency (MAF) >0.01 and high imputation quality (INFO >0.8 and *R*^2^ > 0.5 from IMPUTE2 and Minimac, respectively; see ESM Methods for software details) were included in the analysis.

### GWAS and meta-GWAS of cross-sectional C-peptide

SNPs were tested for association with C-peptide (natural log transformed) under an additive genetic model using Tobit models to appropriately model the large number of participants with values at the lower limit of detection (ESM Table 3) [30]. The Tobit model beta coefficient can be interpreted as the relationship between the genotype and the uncensored latent (unobserved) C-peptide variable. VGLM models from the VGAM library v0.9-7 were fitted using R v3.1.0 [31]. Sex, age at diagnosis and diabetes duration were included as covariates in the model. For fasting C-peptide analysis in the DCCT, the cohort (primary vs secondary) was also included as a covariate in the model.

Meta-GWAS analysis of stimulated C-peptide was performed using METAL v1.5 (www.sph.umich.edu/csg/abecasis/Metal/index.html) with the STDERR method, which weights effect-size estimates by the inverse of the corresponding SEs. However, because C-peptide was measured using different assays in the different studies, and effect-size estimates and standard errors were in different units, the other meta-GWAS analyses used the SAMPLESIZE method, which converts the SNP association test *p* values to *Z* scores using the direction of effect, and combines *Z* scores across studies weighting by the study-specific sample size. Between-study heterogeneity was tested using Cochran’s *Q* statistic. *I*^2^, which depends on *Q*, is used to quantity the level of heterogeneity among studies [32].

### Testing for the heterogeneity of the SNP effect between stimulated and fasting C-peptide

To investigate whether the SNP associations differed between stimulated and fasting C-peptide in the DCCT, we used the Tobit model implementation in the Zelig v 4.2-1 library in R v3.1.0 [31] to account for within-individual correlations of the two C-peptide measurements. Sex, age at diagnosis, diabetes duration, cohort (primary vs secondary) and an indicator for fasting/stimulated C-peptide were included as covariates in the models. Heterogeneity was tested using an interaction between the fasting/stimulated indicator and SNP.

### Type 1 diabetes loci

We investigated two sets of type 1 diabetes loci derived from Oram et al [17] and Onengut-Gumuscu et al [14]. The study of Oram et al included robustly associated genetic variants in both MHC and non-MHC regions from published studies (*n* = 30 SNPs) [33–36], whereas Onengut-Gumuscu et al included only non-MHC regions and was based on Immunochip data in >9000 participants with type 1 diabetes and >12,000 non-diabetic control participants with multiple independent variants in some loci (*n* = 51 SNPs) (see ESM Methods for details).

We stratified SNPs according to the presence (*n* = 70 SNPs) or absence of a prior association with type 1 diabetes, and performed false discovery rate (FDR) control separately for each stratum using the stratified (S)FDR method [37, 38].

### MHC imputation

Classical HLA alleles, their amino acid sequences and additional SNPs in the MHC region were imputed using genotyped SNPs from Illumina 1M BeadArrays in DCCT and Illumina HumanCoreExome BeadArrays in the Joslin 50-Year Medalist, CACTI, WESDR and EDC studies; and SNP2HLA (http://software.broadinstitute.org/mpg/snp2hla). The Type 1 Diabetes Genetic Consortium (T1DGC) dataset (5196 unrelated individuals, including 4323 European participants: 182 participants with type 1 diabetes, 4101 control participants and 182 participants with missing case/control status [39]) was used as the reference panel. The T1DGC panel contains 5868 SNPs (genotyped using the Illumina Immunochip) and four-digit classical HLA types (*HLA-A*, *-B*, *-C*, *-DPA1*, *-DPB1*, *-DQA1*, *-DQB1* and *-DRB1*) [40].

Each of the classical HLA alleles, their amino acid sequences and SNPs in the MHC region were tested for associations with C-peptide, and combined through meta-analysis (as described above).

### Longitudinal stimulated C-peptide in the DCCT

Associations of identified SNPs with repeated measures of stimulated C-peptide (natural log transformed) were tested under an additive genetic model using linear mixed models (random slope and random intercept) and first-order autoregressive AR(1) correlation structure in R v3.1.0 [31]. SNP, sex, age at diagnosis, diabetes duration at baseline, treatment group (intensive vs conventional), cohort (primary vs secondary), an indicator for baseline (vs later assessment), time (DCCT follow-up year), (centred time)^2^; and interactions between time and treatment, treatment and baseline, and SNP and time were included in the model. Differences among SNP genotypes in the rate of C-peptide decline over time were investigated by hypothesis testing of SNP and time interaction.

## Results

### Meta-GWAS

Characteristics of the participants included in stimulated (*n* = 1303), fasting (*n* = 2019), random (*n* = 1497), and stimulated/fasting/random (*n* = 3479) C-peptide meta-GWAS are summarised in ESM Tables 4, 5. C-peptide distributions in different studies are shown in ESM Figs 1, 2.

#### 1q53

**Fig. 2:**
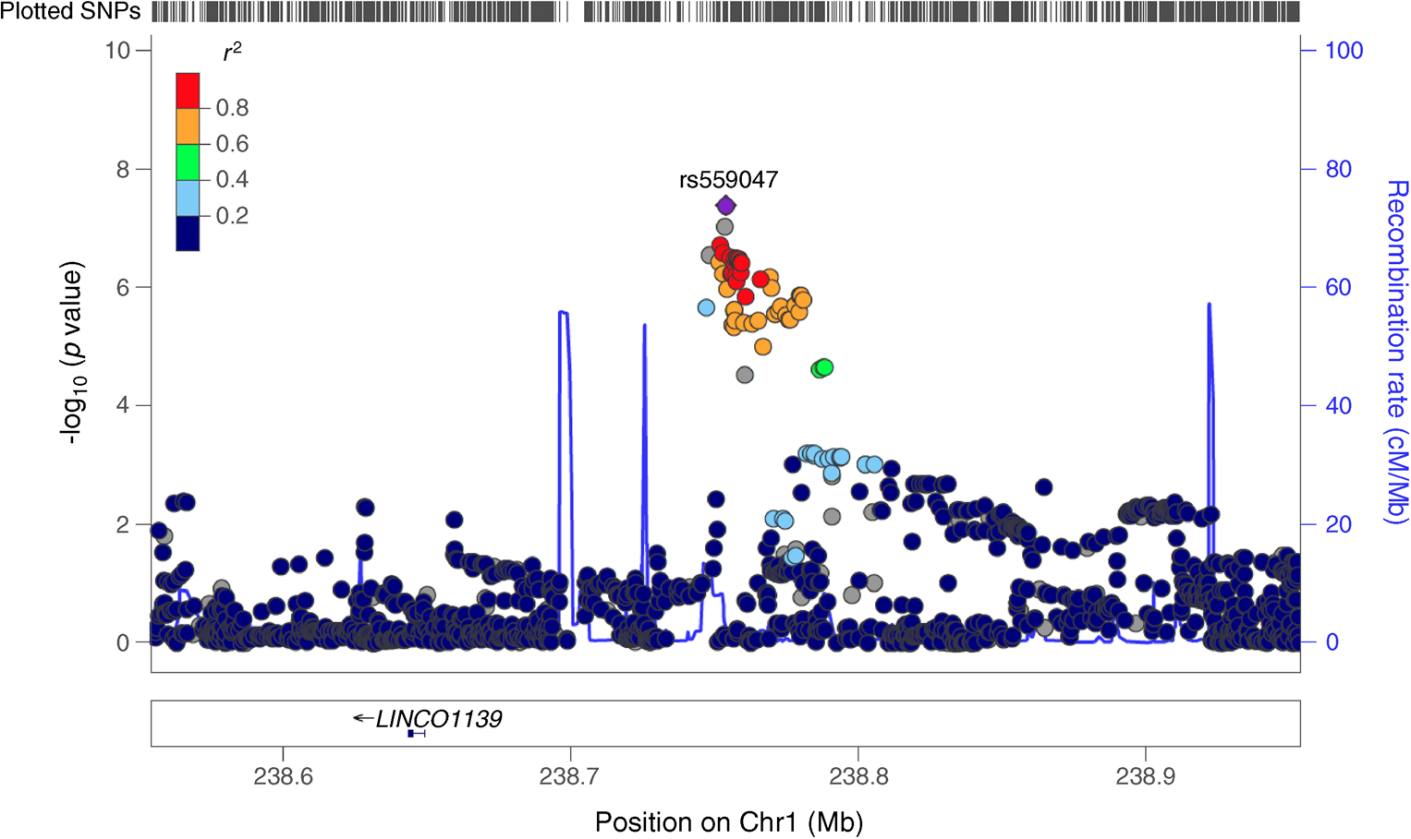
Stimulated/fasting/random C-peptide meta-GWAS, which includes stimulated C-peptide results from the DCCT primary cohort, the secondary cohort with diabetes duration 1–5 years and the secondary cohort with diabetes duration 5–15 years; fasting C-peptide results from the CACTI and EDC studies; and random C-peptide results from the Joslin 50-Year Medalist study and WESDR. The left *y*-axis shows SNP *p* values from the stimulated/fasting/random C-peptide meta-GWAS. The right *y*-axis shows estimated recombination rates. The *x*-axis shows SNP genomic position (Genome Reference Consortium Human Build 37 [GRCh37]/hg19); the LD measures are based on 1000 Genomes Nov 2014 EUR population. The plot was created using LocusZoom (http://locuszoom.sph.umich.edu/locuszoom) [44]. Chr, chromosome

In the stimulated/fasting/random C-peptide meta-GWAS, rs559047 on 1q53 (Chr1: 238753916, T>A) exceeded the genome-wide significance threshold (*p* < 5 × 10^−8^)(Fig. 1; ESM Figs 3, 4). The A allele was associated with lower C-peptide (*p* = 4.13 × 10^−8^). The direction of the rs559047 association with C-peptide was consistent in all studies (Table 1, Fig. 2).

**Table 1:** Association of rs559047 (Chr1: 238753916, T>A) with C-peptide in different studies

Analysis/study	INFO/*R*^2^	MAF	β	SE	*p*	Het *I*^2^	Het *p*
Stimulated C-peptide							
DCCT, primary	0.98	0.24	−0.25	0.07	8.87 × 10^−4^		
DCCT, secondary (duration 1–5 years)	0.98	0.24	−0.44	0.16	0.007		
DCCT, secondary (duration 5–15 years)	0.98	0.24	−0.27	0.13	0.041		
Meta-GWAS			−0.28	0.06	3.74 × 10^−6^	1.18	0.55
Fasting C-peptide							
DCCT	0.96	0.23	−0.13	0.05	0.014		
CACTI	0.95	0.24	−0.6	0.35	0.092		
EDC	0.96	0.25	−1.04	0.86	0.227		
Meta-GWAS					1.36 × 10^−3^	0	0.94
Random C-peptide							
Joslin 50-Year Medalist	0.95	0.25	−0.09	0.04	0.029		
WESDR	0.96	0.26	−0.62	0.36	0.088		
Meta-GWAS					5.60 × 10^−3^	0	0.97
Stimulated/fasting/random C-peptide meta-GWAS					4.13 × 10^−8^	0	0.61

INFO/*R*^2^ indicates the quality of imputation

Alleles are non-effect allele > effect allele

C-peptide was measured with different assays in pmol/ml in the DCCT, Joslin 50-Year Medalist study and WESDR; and in pmol/l in the CACTI and EDC studies; it was also natural log transformed

β, β coefficient; Het, heterogeneity

At DCCT eligibility, fasting and stimulated C-peptide levels were highly positively correlated (Spearman correlation coefficient 0.88, *p* < 0.0001). rs559047 was associated with both stimulated and fasting C-peptide at DCCT eligibility, but there was a significant difference in the association between stimulated and fasting C-peptide (*p* = 0.010), with a larger effect on stimulated compared with fasting C-peptide (−0.28 vs −0.13). The association of rs559047 with stimulated C-peptide remained significant after including fasting C-peptide in the model but not vice versa, suggesting that the rs559047 association with fasting C-peptide is indirect and due to its association with stimulated C-peptide (Table 2).

**Table 2:** Association of the top SNPs and the SNP tagging HLA-A*24 with stimulated C-peptide adjusted for fasting C-peptide and vice versa at DCCT eligibility

Outcome	Covariate	rs9260151 (C>T) Chr6:29911030			rs1264813 (C>T) Chr6:29939900			rs61211515 (T/–) Chr6:30100975			rs3135002 (C>A) Chr6:32668439			rs559047 (T>A) Chr1:238753916		
		β	SE	*p*	β	SE	*p*	β	SE	*p*	β	SE	*p*	β	SE	*p*
S C-peptide	–	0.33	0.09	2.69 × 10^−4^	−0.33	0.08	5.55 × 10^−5^	−0.39	0.07	5.25 × 10^−8^	0.33	0.15	0.031	−0.29	0.06	1.28 × 10^−6^
S C-peptide	F C-peptide	0.21	0.06	1.09 × 10^−3^	−0.26	0.06	1.02 × 10^−5^	−0.31	0.05	1.84 × 10^−9^	0.02	0.11	0.82	−0.19	0.04	5.10 × 10^−6^
F C-peptide	–	0.17	0.08	0.032	−0.19	0.08	0.012	−0.24	0.07	3.46 × 10^−4^	0.35	0.14	9.50 × 10^−3^	−0.17	0.06	1.42 × 10^−3^
F C-peptide	S C-peptide	0.07	0.04	0.12	−0.03	0.04	0.42	−0.06	0.04	0.098	0.08	0.07	0.25	0.02	0.03	0.61

Alleles are non-effect allele > effect allele

Associations were tested using QLIM procedure in SAS v9.4 (SAS, Cary, NC, USA), and are based on extracted genotypes from dosage data according to the best guess using GTOOL v0.7.5 (www.well.ox.ac.uk/~cfreeman/software/gwas/gtool.html) adjusting for sex, age at diagnosis, type 1 diabetes duration and cohort (primary vs secondary)

β, β coefficient; F, fasting; S, stimulated

rs559047 was not associated with the rate of decline in stimulated C-peptide over time using longitudinal DCCT data. Characteristics of the participants included in the DCCT longitudinal data analysis are summarised in ESM Table 6.

#### MHC region

Multiple variants in the MHC region were associated with different C-peptide measures. In the stimulated/fasting/random C-peptide meta-GWAS, two loci in the MHC region, rs3135002 (Chr6:32668439) and rs9260151 (Chr6:29911030), approximately 2.8 Mb apart from each other, were close to genome-wide significance. A single nucleotide deletion in the MHC region, rs61211515 (Chr6:30100975), had the lowest *p* value in the stimulated C-peptide meta-GWAS (Fig. 1; ESM Figs 3, 4). Detailed results for these three loci follow.

#### rs3135002

**Fig. 3:**
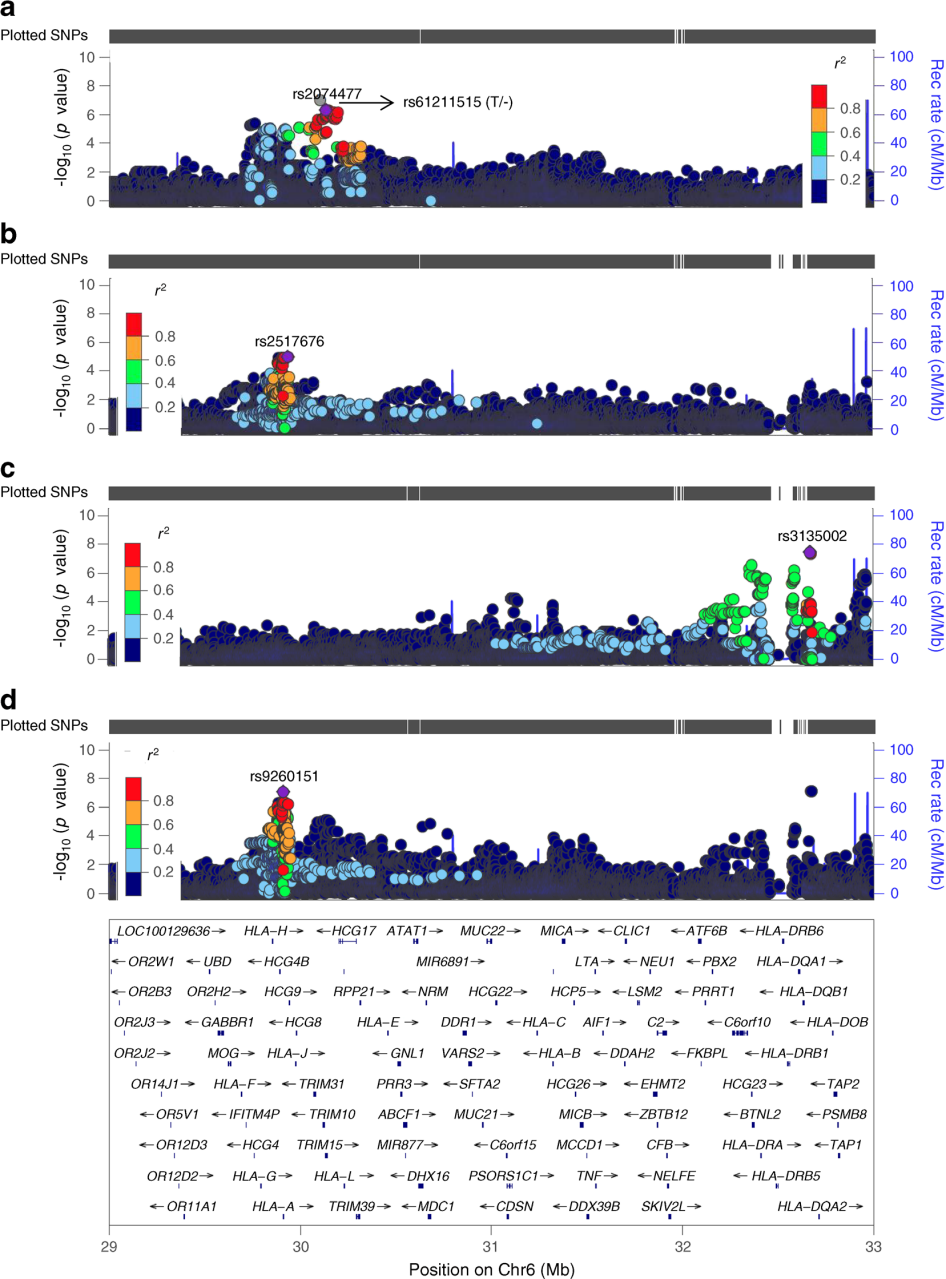
(**a**) Stimulated C-peptide meta-GWAS, which includes the DCCT primary cohort, the secondary cohort with diabetes duration 1–5 years and the secondary cohort with diabetes duration 5–15 years. Since rs61211515 (T/–) is an insertion/deletion (in/del) second-to-top hit, rs2074477, in high LD with rs61211515 (*r*^2^ 0.89; *D*′ 0.98) was used by LocusZoom for LD measures. (**b**) Fasting C-peptide meta-GWAS, which includes the DCCT, CACTI and EDC studies. (**c**) Random C-peptide meta-GWAS, which includes the Joslin 50-Year Medalist study and WESDR. (**d**) Stimulated/fasting/random C-peptide meta-GWAS, which includes stimulated C-peptide results from the DCCT primary cohort, the secondary cohort with diabetes duration 1–5 years and the secondary cohort with diabetes duration 5–15 years; fasting C-peptide results from the CACTI and EDC studies; and random C-peptide results from the Joslin 50-Year Medalist study and WESDR. Left *y*-axis: SNP *p* values; right *y*-axis: estimated recombination rates; *x*-axis: SNP genomic position (Genome Reference Consortium Human Build 37 [GRCh37]/hg19); the LD measures are based on the 1000 Genomes November 2014 EUR population. The plot was made using LocusZoom (http://locuszoom.sph.umich.edu/locuszoom/) [44]. Chr, chromosome; Rec, recombination

rs3135002 (C>A) exceeded the genome-wide significance threshold in the random C-peptide meta-GWAS (*p* = 3.49 × 10^−8^). The A allele was associated with higher random C-peptide in both the Joslin 50-Year Medalist study (β [SE] = 0.44 [0.12], *p* = 3.22 × 10^−4^) and the WESDR (β [SE] = 2.34 [0.54], *p* = 1.53 × 10^−5^). rs3135002 was also associated with fasting C-peptide at DCCT eligibility (β [SE] = 0.36 [0.13], *p* = 5.42 × 10^−3^). Although it was not associated with stimulated C-peptide in the DCCT (β [SE] = 0.27 [0.15], *p* = 0.077), the effect was in the same direction; further, there was no significant difference between the fasting and stimulated C-peptide associations (*p* = 0.46). rs3135002 was not associated with fasting C-peptide in the CACTI or EDC studies, and nor was it associated with the rate of stimulated C-peptide decline over time in the DCCT (*p* = 0.62) (Table 3, Fig. 3).

**Table 3:** Association of SNPs in the MHC region with C-peptide in different studies

Analysis/study	*n*	rs9260151 (Chr6:29911030; C>T)					rs61211515 (Chr6:30100975; T/–)					rs3135002 (Chr6:32668439; C>A)				
		INFO/*R*^2^	MAF	β	SE	*p* Het *I*^2^ (*p*)	INFO/*R*^2^	MAF	β	SE	*p* Het *I*^2^ (*p*)	INFO/*R*^2^	MAF	β	SE	*p* Het *I*^2^ (*p*)
Stimulated C-peptide																
DCCT, primary	651	0.96	0.09	0.26	0.11	0.016	0.91	0.16	−0.34	0.09	1.13 × 10^−4^	0.97	0.03	0.2	0.19	0.294
DCCT, secondary (duration 1–5 years)	135	0.96	0.09	0.71	0.28	0.011	0.91	0.17	−0.36	0.21	0.084	0.97	0.03	0.33	0.41	0.423
DCCT, secondary (duration 5–15 years)	517	0.96	0.09	0.44	0.19	0.020	0.91	0.16	−0.59	0.17	4.69 × 10^−4^	0.97	0.03	0.39	0.3	0.193
Meta-GWAS				0.34	0.09	1.02 × 10^−4^ 23.9 (0.27)			−0.39	0.07	9.72 × 10^−8^ 1.70 (0.43)			0.27	0.15	0.077 0 (0.86)
Fasting C-peptide																
DCCT	1340	0.97	0.09	0.2	0.08	0.012	0.86	0.18	−0.19	0.07	3.53 × 10^−3^	0.98	0.06	0.36	0.13	5.42 × 10^−3^
CACTI	529	0.97	0.09	1.35	0.41	0.001	0.88	0.17	0.34	0.38	0.368	0.91	0.03	1.07	0.77	0.163
EDC	150	0.94	0.07	2.50	1.09	0.021	0.88	0.19	0.05	0.86	0.959	0.96	0.03	0.97	1.77	0.586
Meta-GWAS						1.21 × 10^−5^ 41.6 (0.18)					0.057 64.9 (0.058)					1.77 × 10^−3^ (0.91)
Random C-peptide																
Joslin 50-Year Medalist study	906	0.97	0.1	0.08	0.06	0.197	0.98	0.17	−0.10	0.05	0.065	0.94	0.02	0.44	0.12	3.22 × 10^−4^
WESDR	591	0.97	0.09	0.56	0.47	0.237	0.88	0.18	−0.94	0.45	3.49 × 10^−2^	0.93	0.03	2.34	0.54	1.53 × 10^−5^
Meta-GWAS						0.081 0 (0.91)					5.73 × 10^−3^ 0 (0.63)					3.49 × 10^−8^ 18 (0.27)
Stimulated/fasting/random																
C-peptide meta-GWAS						8.43 × 10^−8^ 27.8 (0.22)					1.51 × 10^−6^ 61.7 (0.016)					7.19 × 10^−8^ 27.7 (0.22)

INFO/*R*^2^ indicates the quality of imputation

Alleles are non-effect allele > effect allele

C-peptide was measured with different assays in pmol/ml in the DCCT, Joslin 50-Year Medalist study and WESDR; and in pmol/l in the CACTI and EDC studies; it was also natural log transformed

β, β coefficient; Het, heterogeneity

#### rs9260151

rs9260151 (C>T) is an intronic SNP within *HLA-A*. Its T allele was associated with higher C-peptide with a *p* value close to the genome-wide significance threshold in the stimulated/fasting/random C-peptide meta-GWAS (*p* = 8.43 × 10^−8^). The direction of the effect was consistent in all studies (Table 3, Fig. 3). rs9260151 was associated with both stimulated and fasting C-peptide at DCCT eligibility but the effects were significantly different (*p* = 0.024), with a larger effect on stimulated compared with fasting C-peptide (0.34 vs 0.20). Its association with stimulated C-peptide remained significant after including fasting C-peptide in the model, but not vice versa (Table 2). rs9260151 was not associated with differences in the rate of decline in stimulated C-peptide over time using longitudinal DCCT data.

#### rs61211515

A single nucleotide deletion in the MHC region, rs61211515 (T/–), had the lowest *p* value (β [SE] = −0.39 [0.07], *p* = 9.72 × 10^−8^) in the stimulated C-peptide meta-GWAS (Table 3, Fig. 3). rs61211515 was also associated with fasting C-peptide at DCCT eligibility (β [SE] = −0.23 [0.07], *p* = 6.80 × 10^−4^). The association of rs61211515 (T/–) with stimulated C-peptide remained significant after including fasting C-peptide in the model (β [SE] = −0.28 [0.05], *p* = 2.77 × 10^−8^), whereas its association with fasting C-peptide became insignificant after including stimulated C-peptide in the model (*p* = 0.10) (Table 2). The strength of rs61211515 association with fasting and stimulated C-peptide differed significantly (*p* = 4.66 × 10^−3^), with a larger effect on stimulated compared with fasting C-peptide (−0.39 vs −0.23). rs61211515 (T/–) was not significantly associated with fasting C-peptide in the CACTI or EDC studies, but it was associated with random C-peptide in WESDR with the same direction of effect (β [SE] = −0.94 [0.45], *p* = 0.035). Although its association with random C-peptide was not significant in the Joslin 50-Year Medalist study (β [SE] = −0.10 [0.05], *p* = 0.065), the effect direction was the same (Table 3). The rate of decline in stimulated C-peptide over time in the DCCT depended on the rs61211515 (T/−) genotype (SNP–time interaction β [SE] = −0.08 [0.04], *p* = 0.023). Association of rs61211515 with stimulated C-peptide at DCCT eligibility in participants with and without longitudinal data is shown in ESM Table 7. rs61211515 is a mononucleotide T repeat (*n* = 12 Ts in RefSeq (https://www.ncbi.nlm.nih.gov/refseq/, accessed 1 July, 2017)). An insertion allele at the same locus (T/TT) was not associated with stimulated, fasting or random C-peptide; and comparing five genotype categories with a homozygous reference, only categories with deletion had significant effects (ESM Tables 8, 9).

#### Independence of the MHC signals

rs9260151, rs3135002 and rs61211515 are in linkage disequilibrium (LD) (ESM Table 10). However, putting them in the same model, all three remained significantly associated with stimulated and fasting C-peptide at DCCT eligibility (ESM Table 11). The likelihood ratio test comparing a model with no SNPs (only covariates in the model and stimulated C-peptide as the outcome) to a model with all three SNPs (rs61211515, rs9260151, rs3135002 and covariates) was strongly significant (*p* = 5.67 × 10^−10^).

#### MHC imputation

A total of 424 classical HLA alleles were imputed: 279 were polymorphic, and of them 252 had good imputation quality (*R*^2^ > 0.5) in the DCCT. Similarly, 1136/1276 amino acid changes were polymorphic, and 1106 met imputation quality criteria. A total of 7144/7261 SNPs were polymorphic and 7066 were imputed well (ESM Table 12). None of the HLA classical alleles or amino acid changes reached the genome-wide significant threshold in the meta-GWAS.

### Association of type 1 diabetes loci and C-peptide

Five type 1 diabetes risk loci were nominally associated (*p* < 0.05) with lower stimulated C-peptide at DCCT eligibility: rs1264813 tagging HLA-A*24 (β [SE] = −0.34 [0.08], *p* = 2.80 × 10^−5^), rs151234 (*IL27*) (β [SE] = −0.32 [0.08], *p* = 3.15 × 10^−5^), rs12971201 (*PTPN2*) (β [SE] = −0.13 [0.05], *p* = 9.38 × 10^−3^), rs689 (*INS*) (β [SE] = −0.18 [0.07], *p* = 0.011), and rs193778 (*DEXI*) (β [SE] = −0.11 [0.06], *p* = 0.044). Association of these SNPs with fasting/random C-peptide in the other studies is shown in ESM Table 13.

The SFDR method estimated the proportion of null SNPs (*π*_0_) at 0.91 in the prior type 1 diabetes stratum vs 0.99 in the stratum of the remaining SNPs. Setting an FDR control level of 20% yielded four test rejections for prior type 1 diabetes SNPs and no test rejections in the remaining SNPs. The FDR *q* value estimates were 0.001 for both rs1264813 and rs151234, and 0.17 for both rs12971201 and rs689. SFDR ranked these four SNPs above rs61211515, the top associated SNP in the stimulated C-peptide meta-GWAS.

The association of rs1264813 (HLA-A*24) with stimulated C-peptide at DCCT eligibility did not remain significant when rs61211515 (the top GWAS-associated SNP in this region) was included in the model (*p* = 0.45). Similar results were observed with imputed HLA-A*24; it was associated with lower stimulated C-peptide at DCCT eligibility (β [SE] = −0.39 [0.09], *p* = 1.50 × 10^−5^), but this association became insignificant after adjustment for rs61211515 (*p* = 0.09) (ESM Table 14). rs1264813 (HLA-A*24) was also nominally associated with fasting C-peptide at DCCT eligibility (β [SE] = −0.19 [0.07], *p* = 0.013) but its association with stimulated C-peptide was significantly stronger (*p* = 0.011). The rs1264813 association with stimulated C-peptide remained significant when fasting C-peptide was included in the model but not vice versa (Table 2). rs1264813 was also associated with an increased rate of decline in stimulated C-peptide over time in the DCCT (SNP–time interaction β [SE] = −0.11 [0.04], *p* = 6.32 × 10^−3^).

*HLA-DR3* (β [SE] = 0.03 [0.06], *p* = 0.59) and *HLA-DR4-DQ8* (β [SE] = 0.06 [0.06], *p* = 0.29) were not associated with stimulated C-peptide at DCCT eligibility*. HLA-DR3*/*DR4-DQ8* compound heterozygotes had higher stimulated C-peptide compared with individuals having no copy of *DR3* or *DR4-DQ8* (β [SE] = 0.26 [0.11], *p* = 0.024). However, this association did not withstand multiple testing correction (ESM Table 15).

*HLA-DQB1* position 57, *HLA-DRB1* position 13 or *HLA-DRB1* position 71 amino acid variants, which have been reported to independently drive type 1 diabetes risk and explain >90% of the type 1 diabetes–HLA association [41], were not associated with stimulated C-peptide at DCCT eligibility.

Of the two type 1 diabetes GRS, only the Onengut-Gumuscu et al type 1 diabetes GRS was nominally associated with lower stimulated C-peptide (β [SE] = −0.09 [0.04], *p* = 0.036) at DCCT eligibility. This association did not remain significant after excluding rs151234 (*IL27*); β [SE] = −0.07 [0.04], *p* = 0.085.

## Discussion

By combining GWAS data from five type 1 diabetes studies including approximately 1300 individuals with stimulated and approximately 3500 individuals with stimulated, fasting or random C-peptide data, we identified association with a locus on chromosome 1 (rs559047) and multiple independent variants in the MHC region.

rs559047 is an intergenic SNP located 700 kb from the nearest gene (*ZP4*). It is not a significant *cis*-expression quantitative trait locus (*cis*-eQTL) for nearby genes in any tissue, including the pancreas (www.gtexportal.org, accessed 14 July 2017) or pancreatic islets (http://theparkerlab.org/tools/isleteqtl, accessed 14 July 2017). However, the *cis*-eQTLs were produced using a limited number of samples (149 and 112 for pancreas and islets, respectively) from non-diabetic individuals. rs559047 has not been associated with the risk of type 1 diabetes (ESM Table 16), and variants in this region have not previously been associated with type 1 diabetes related phenotypes.

We identified three SNPs (rs9260151, rs61211515 and rs3135002) in the MHC region associated with C-peptide levels. Of these, rs9260151 and rs3135002 have been associated with risk of type 1 diabetes (ESM Table 16). rs9260151 is an intronic SNP within *HLA-A*, while rs61211515 is located approximately 187 kb downstream of *HLA-A*. Both SNPs appear to be primarily associated with stimulated C-peptide although rs9260151 was identified in the meta-GWAS of stimulated/fasting/random C-peptide, likely due to the larger available sample size. HLA-A*24 is a known risk variant for type 1 diabetes [34, 42]. However, after including rs9260151, rs61211515 and HLA-A*24 (either imputed or tagging SNP) in the model, rs9260151 and rs61211515 both remained significantly associated with C-peptide, indicating that they are independent signals and distinct from HLA-A*24. The association between HLA-A*24 and C-peptide was no longer significant, suggesting that this association is due to LD between HLA-A*24 and rs9260151 and/or rs61211515 (ESM Table 10). Similarly, the association of rs9260151 with type 1 diabetes could be explained by its LD with HLA-A*24. rs61211515 and HLA-A*24, which were associated with lower C-peptide levels, were also associated with an accelerated decline in C-peptide levels over time, providing more evidence that this association is a true finding. HLA-A*24 has previously been associated with undetectable C-peptide in a Japanese type 1 diabetes population sample [15] and with more rapid beta cell destruction post islet transplantation [43]. The third SNP, rs3135002, is intergenic between *HLA-DQB1* and *DQA2*, 2.5 Mb from rs9260151 and rs61211515. Its association with C-peptide remained significant when both rs9260151 and rs61211515 were included in the model, indicating that it is independent. *DR3* and *DR4-DQ8* were not associated with C-peptide and the borderline association observed between *DR3*/*DR4-DQ8* compound heterozygotes and C-peptide did not withstand multiple testing correction, suggesting that this effect is also distinct from the *DR3*/*DR4-DQ8* haplotype, consistent with previous findings [15]. The association of rs3135002 with type 1 diabetes could be explained by the extremely large effect of *DR3*/*DR4-DQ8* on type 1 diabetes and the LD between rs3135002 and both *DR3* (*D*′ = 1, *r*^2^ = 0.012) and *DR4-DQ8* (*D*′ = 0.87, *r*^2^ = 0.010). The rs3135002 association was mainly with fasting/random C-peptide rather than with stimulated C-peptide. No significant interactions were observed between the three SNPs in the MHC affecting stimulated/fasting C-peptide (data not shown), and none of them was a significant *cis*-eQTL for the nearby genes in any tissue including the pancreas and pancreatic islets (http://www.gtexportal.org, accessed July 14, 2017; http://theparkerlab.org/tools/isleteqtl, accessed July 14, 2017).

Some SNP associations for C-peptide in type 1 diabetes are different from those for type 1 diabetes. Two of the identified variants (rs559047 on chromosome 1 and rs61211515 in the MHC region) have not been previously associated with type 1 diabetes; while rs9260151 and rs3135002 in the MHC region have been associated with type 1 diabetes. The variants associated with C-peptide but not with type 1 diabetes may affect gene expression, and fine-mapping and functional studies are required to determine their effects. However, SFDR analysis suggests that the proportion of type 1 diabetes loci associated with C-peptide is greater than for the rest of the genome; four out of 70 type 1 diabetes loci (HLA-A*24, *IL27*, *INS* and *PTPN2*) that were nominally associated with lower C-peptide levels ranked higher than rs61211515, the top associated SNP in the stimulated C-peptide meta-GWAS. Nevertheless, the composition and magnitude of SNP effects for type 1 diabetes development vs progression could differ, explaining why there is no significant association between type 1 diabetes GRS and C-peptide.

Some limitations include that only fasting/random C-peptide measurements were available for about 60% of individuals, whereas stimulated C-peptide is more appropriate to evaluate insulin secretion in type 1 diabetes. In addition, individuals from different cohorts were quite different in terms of their age at diagnosis, diabetes duration and other inclusion/exclusion criteria, all of which may influence C-peptide levels. C-peptide can also be measured with different methods with different lower limits of detection, and these were not standardised among the studies. The mean age at diagnosis ranged from 8.3 years in the EDC study to 21.2 years in the DCCT, and the mean type 1 diabetes duration ranged from 5.6 years in the DCCT to 54.7 years in the Joslin 50-Year Medalist study. Participants from the DCCT, the only study with stimulated C-peptide data, were highly selected as they were diagnosed with type 1 diabetes later in life and had diabetes for a shorter period of time. Therefore, the DCCT had a larger proportion of participants with detectable C-peptide compared with the other studies. This heterogeneity among the cohorts might have reduced the power of the analysis and contributed to non-replication at specific loci.

In conclusion, we have identified a locus on chromosome 1 and multiple variants in the MHC region that are associated with C-peptide levels. However, these associations need to be validated in independent populations. Further investigations could eventually provide insight into mechanisms of beta cell loss and opportunities to preserve beta cell function.

## Electronic supplementary material

ESM (PDF 843 kb)

## Data Availability

DCCT data are available to authorised users at https://repository.niddk.nih.gov and www.ncbi.nlm.nih.gov/projects/gap/cgi-bin/study.cgi?study_id=phs000086.v3.p1. For Joslin 50-Year Medalist, CACTI, WESDR and EDC studies, the data are available on request from the authors.

## Abbreviations

CACTI: Coronary Artery Calcification in Type 1 Diabetes
Chr: Chromosome
EDC: Pittsburgh Epidemiology of Diabetes Complications
eQTL: Expression quantitative trait loci
FDR: False discovery rate
GRS: Genetic risk score
GWAS: Genome-wide association study
LD: Linkage disequilibrium
MAF: Minor allele frequency
SFDR: Stratified false discovery rate
T1DGC: Type 1 Diabetes Genetics Consortium
WESDR: Wisconsin Epidemiologic Study of Diabetic Retinopathy
