# Supplementary material for: Meta-genome-wide association studies identify a locus on chromosome 1 and multiple variants in the MHC region for serum C-peptide in type 1 diabetes
Source: Diabetologia. 2018 Feb 5;61(5):1098–111. doi: 10.1007/s00125-018-4555-9 (PMC5876265; doi:10.1007/s00125-018-4555-9)
Supplement: Supplementary file 1 — (PDF 843 kb) [file 125_2018_4555_MOESM1_ESM.pdf]

ELECTRONIC SUPPLEMENTARY MATERIAL

SUPPLEMENTARY METHODS

Genotyping, Quality Control and Imputation:

Genotyping was performed using both Illumina 1M [1] and HumanCoreExome-12 v1.1 BeadArrays (Illumina, San Diego, CA, USA) in DCCT; Illumina HumanCoreExome-24 BeadArrays (v1.0) in Medalist; and Illumina HumanCoreExome-12 BeadArrays in CACTI (v1.0), WESDR (v1.0) and EDC (v1.1).

Details of genotyping and quality control regarding Illumina 1M bead array in DCCT were published previously [1].

HumanCoreExome genotyping, quality control and imputation for all five studies (DCCT, CACTI, EDC, Joslin 50 Year Medalist and WESDR) were performed in a single site by one laboratory and one analytic team at the University of Virginia (UVA). SNPs with 20% (or 5% for rare variants) MAF difference with 1000 Genomes were removed. SNP and subject call rates were set to 95% and 98%, respectively. SNPs deviated from Hardy-Weinberg equilibrium ($p < 1E-6$) were excluded. Ten principal components (PCs) were calculated and outliers according to PC analysis (PCA) were excluded. To detect cryptically related individuals, identical-by-state estimates between all pairs of individuals were performed, and one from each pair was excluded.

SHAPEIT v2 (r837) is used for haplotype phasing [2, 3]. Autosomal SNPs ungenotyped on Illumina 1M and HumanCoreExome BeadArrays were imputed using the 1000 Genomes data phase3, v5 (updated on Oct 20, 2015)[4]; and IMPUTE2 v.2.3.0 (https://mathgen.stats.ox.ac.uk/impute/impute_v2.html)[5] and Minimac3 v1.0.13 (updated on Oct 15, 2015)(http://csg.sph.umich.edu/abecasis/MaCH/index.html)[6, 7], respectively.

All SNP coordinates mentioned in the paper are based on Human hg19.

Type 1 Diabetes (T1D) Genetic Risk Score (GRS):

SNP genotypes regarding both sets of T1D loci derived from Oram et al [8] and Onengut-Gumuscu et al [9] were extracted from dosage data according to the best guess using GTOOL, version 0.7.5 (http://www.well.ox.ac.uk/~cfreeman/software/gwas/gtool.html). Subsequently, T1D GRS was generated by summing, across all SNPs, the product of the number of risk-increasing alleles (0, 1, or 2) at each SNP and the corresponding natural log (OR) [8].

The *DR3* (*DRB1*0301-DQA1*0501-DQB1*0201*) and *DR4-DQ8* (*DRB1*04-DQA1*0301-DQB1*0302*) haplotypes do not fit this log-additive model, with *DR3*/*DR4-DQ8* individuals having the highest odds ratio. Therefore, weights for *DR3*/*DR4-DQ8* were assigned based on imputed haplotypes, and odds ratios from Winkler et al [8, 10, 11].

Association of each of T1D GRS, *DR3*/*DR4* GRS, and six *DR3*/*DR4* genotype categories (*DR3*/*DR3*, *DR3*/*DR4*, *DR4*/*DR4*, *DR3*/X, X/*DR4*, X/X) with stimulated C-peptide at DCCT eligibility was tested using Tobit models (QLIM procedure, SAS version 9.4 (SAS, Cary, NC)). The Primary Cohort, Secondary Cohort with duration 1-5 years, and Secondary Cohort with duration 5-15 years were analyzed

separately, adjusting for sex, age at diagnosis and duration; and the results were combined though meta-analysis. The association of each genetic factor with fasting C-peptide at DCCT eligibility was also tested in all subjects further adjusting for Cohort (Primary vs. Secondary).

The association of T1D GRS and DR3/DR4 GRS with rate of decline in stimulated C-peptide over time in DCCT was investigated using linear mixed models (as described in the main text).

SUPPLEMENTARY TABLES

ESM Table 1: Characteristics of the DCCT subjects and their first-degree affected relatives included in the family study

	**Proband (N = 98)**	**First-degree* Affected Relative (N = 109)**
Sex (Male)	52 (53.1%)	58 (53.2%)
Age at Diagnosis (Years)	21.1 (7.4)	22.0 (14.5)
T1D Duration (Years)	5.9 (4.2)	16.3 (10.1)
Detectable Stimulated C-peptide (>0.03 pmol/mL)	52 (47.7%)	20 (18.4%)

* Sibling or parent
Values are Mean (SD) or N (%)

ESM Table 2: Number of first-degree relatives with non-detectable stimulated C-peptide for probands in the DCCT family study

Stimulated C-peptide in Proband	Stimulated C-peptide in First-Degree Relatives		
	**Undetectable (≤ 0.03 pmol/mL)**	**Detectable (> 0.03 pmol/mL)**	**Total**
**Undetectable (≤ 0.03 pmol/mL)**	48 (84.2%)	9 (15.8%)	57
**Detectable (> 0.03 pmol/mL)**	41 (78.8%)	11 (21.2%)	52
**Total**	89	20	109

Generalized estimating equation (GEE) regression adjusting for relatives' sex, age at diagnosis and T1D duration showed a higher proportion of non-detectable C-peptide in relatives of probands who had non-detectable C-peptide compared to those with detectable C-peptide (OR = 1.45) but precision was insufficient to exclude a chance finding (OR (CI) = (0.48-4.44), p = 0.51).

ESM Table 3: C-peptide measurement methods in different studies

Study	Time	Assay	Lower Limit of Detection
DCCT	After 8-12 hr fasting<br>90 mins after standard meal*	M-1230 antiserum and other reagents obtained from Novo Industri (Bagsvaerd, Denmark)	0.03 pmol/mL
CACTI	After 12 hr fasting	ALPCO C-peptide ELISA Kit (ALPCO, Salem, NH)	16 pmol/L
EDC	After 8 hr fasting	Mercodia Ultrasensitive C-peptide ELISA (Mercodia AB, Uppsala, Sweden)	1.15 pmol/L
Medalist	Non-fasting	Radioimmunoassay (Beckman Coulter, Fullerton, CA)	0.05 pmol/mL
WESDR	Non-fasting	Radioimmunoassay with Heding's M1230 antiserum	0.03 pmol/mL

* 6 mL of Sustacal/kg of body weight to a maximum of 360 mL (Mead-Johnson, Evansville, Indiana; 1 calorie/mL; 55% carbohydrate, 24% protein, and 21% fat) in a period not exceeding 10 minutes

ESM Table 4: Characteristics of the DCCT participants included in the stimulated C-peptide analysis at eligibility

Cohort	Primary	Secondary Duration 1-5 Yrs	Secondary Duration 5-15 Yrs	All
N	651	135	517	1,303
Sex (Male)	341 (52.4%)	83 (61.5%)	271 (52.4%)	695 (53.3%)
Age at T1D Diagnosis (years)	23.9 (7.8)	29.0 (7.3)	17.2 (7.1)	21.2 (8.1)
Duration (Years)	2.6 (1.3)	3.2 (1.3)	10.2 (2.7)	5.7 (4.2)
Oram et al T1D GRS	15.85 (1.53)	16.01 (1.53)	16.05 (1.45)	15.95 (1.50)
Onengut_Gumuscu et al T1D GRS	9.63 (0.85)	9.61 (0.89)	9.61 (0.88)	9.59 (0.87)
Stimulated C-peptide (pmol/mL)				
N with Detectable Values	495 (76.0%)	102 (75.6%)	130 (25.1%)	727 (55.8%)
Not Transformed				
All	0.13 (0.04-0.26)	0.11 (0.04-0.22)	0.03 (0.03-0.04)	0.05 (0.03-0.17)
Subjects with Detectable Values	0.19 (0.10-0.30)	0.16 (0.10-0.30)	0.07 (0.05-0.12)	0.15 (0.08-0.27)
Natural Log Transformed				
All	-2.20 (0.95)	-2.27 (0.92)	-3.26 (0.49)	-2.63 (0.95)
Subjects with Detectable Values	-1.78 (0.68)	-1.87 (0.69)	-2.53 (0.49)	-1.93 (0.71)
Fasting C-peptide (pmol/mL)				
N with Detectable Values	398 (61.1%)	84 (62.2%)	71 (13.7)	553 (42.4%)
Not Transformed				
All	0.06 (0.03-0.11)	0.05 (0.03-0.09)	0.03 (0.03-0.03)	0.03 (0.03-0.07)
Subjects with Detectable Values	0.09 (0.06-0.14)	0.08 (0.06-0.12)	0.05 (0.04-0.07)	0.08 (0.06-0.13)
Natural Log Transformed				
All	-2.81 (0.69)	-2.87 (0.64)	-3.42 (0.25)	-3.06 (0.63)
Subjects with Detectable Values	-2.37 (0.53)	-2.49 (0.51)	-2.87 (0.32)	-2.45 (0.53)

Values are Mean (SD), N (%) or mean (25-75 percentiles).

ESM Table 5: Characteristics of the study participants included in the fasting/random C-peptide analysis

	Fasting C-peptide			Random C-peptide	
**Study**	**DCCT**	**CACTI**	**EDC**	**Medalist**	**WESDR**
N	1,340	529	150	906	591
Sex (Male)	707 (52.8)	235 (44.4)	76 (50.7)	409 (45.1)	297 (50.3)
Age at T1D Diagnosis (years)	21.2 (8.1)	13.1 (8.0)	8.3 (4.1)	11.1 (6.4)	14.5 (7.5)
Duration (Years)	5.6 (4.2)	34.1 (9.0)	43.0 (6.7)	54.7 (5.8)	19.2 (9.0)
N with Detectable C-peptide	572 (42.7%)	83 (15.7%)	14 (9.3%)	304 (33.6%)	68 (11.5%)
C-peptide					
All Subjects	0.03 (0.03-0.07)*	16 (16-16)†	1.15 (1.15-1.15)†	0.05 (0.05, 0.13)*	0.03 (0.03-0.03)*
Subjects with Detectable Values	0.08 (0.06-0.13)*	58.82 (28.00-278.40) †	3.75 (2.69-9.88)†	0.20 (0.13, 0.31)*	0.14 (0.07-0.46)*
C-peptide Natural Log Transformed					
All Subjects	-3.06 (0.63)	3.05 (0.82)	0.28 (0.53)	-2.53 (0.76)	-3.31 (0.64)
Subjects with Detectable Values	-2.45 (0.53)	4.51 (1.32)	1.65 (1.02)	-1.61 (0.67	-1.81 (0.99)

Values are Mean (SD), N (%) or mean (25-75 percentiles).
* pmol/mL
† pmol/L

ESM Table 6: Characteristics of the DCCT participants included in the longitudinal stimulated C-peptide analysis

Study	Eligibility	Year 1	Year 2	Year3	Year 4	Year 5	Year 6
N	258	248	246	200	116	40	21
Sex (Male)	139 (53.9%)	132 (53.2%)	137 (55.7%)	107 (53.5%)	64 (55.2%)	18 (45.0%)	11 (52.4%)
Cohort (Primary)	222 (86.1%)	213 (85.9%)	210 (85.4%)	167 (83.5%)	89 (76.7%)	28 (70.0%)	17 (81.0%)
Treatment Group (Conventional)	137 (53.1%)	132 (53.2%)	131 (53.3%)	102 (51.0%)	59 (50.9%)	18 (45.0%)	10 (47.6%)
Age at T1D Diagnosis (years)	25.9 (6.8)	25.9 (6.9)	25.8 (6.8)	26.1 (6.8)	26.5 (6.2)	25.2 (6.5)	23.1 (8.3)
Duration at Eligibility (Months)	2.1 (1.1)	2.1 (1.1)	2.1 (1.1)	2.2 (1.1)	2.3 (1.1)	2.6 (1.2)	2.5 (1.0)
N with Detectable stimulated C-peptide	258	244	231	179	110	38	13
Stimulated C-peptide							
All Subjects	0.31 (0.26-0.38)	0.19 (0.12-0.31)	0.14 (0.07-0.23)	0.11 (0.07-0.23)	0.13 (0.08-0.22)	0.11 (0.07-0.24)	0.04 (0.03-0.09)
Subjects with Detectable Values	0.31 (0.26-0.38)	0.20 (0.13-0.31)	0.15 (0.09-0.24)	0.13 (0.09-0.24)	0.13 (0.08-0.22)	0.12 (0.07-0.24)	0.09 (0.05-0.10)
Stimulated C-peptide Natural Log Transformed							
All Subjects	-1.16 (0.24)	-1.71 (0.71)	-2.03 (0.79)	-2.13 (0.81)	-2.06 (0.72)	-2.16 (0.77)	-2.89 (0.71)
Subjects with Detectable Values	-1.16 (0.24)	-1.68 (0.67)	-1.94 (0.72)	-1.97 (0.69)	-1.98 (0.65)	-2.09 (0.72)	-2.51 (0.68)

ESM Table 7: Association of rs61211515 (T/-) with stimulated C-peptide at DCCT eligibility based on subjects status of having repeated measures of stimulated C-peptide

Having Repeated Measures of Stimulated C-peptide	N (Detectable/Undetectable)	BETA	SE	P*
Yes	258 (258/0)	0.002	0.031	0.946
No	1,045 (469/576)	-0.459	0.079	6.56E-9
Duration 1-5 Years	520 (339/181)	-0.412	0.089	3.88E-6
Duration 5-15 Years	525 (130/395)	-0.612	0.168	2.58E-4
All	1,303 (727/576)	-0.395	0.72	5.25E-8

Associations were tested using QLIM procedure in SAS version 9.4 (SAS, Cary, NC), and are based on extracted genotypes from dosage data according to the best guess using GTOOL, version 0.7.5 (http://www.well.ox.ac.uk/~cfreeman/software/gwas/gtool.html) adjusting for sex, age at diagnosis, T1D duration and cohort (primary vs. secondary).
* rs61211515 (T/-) association with stimulated C-peptide at DCCT eligibility was significantly heterogeneous between those with values ≤0.20 and >0.20 (p = 0.003).

ESM Table 8: Association of rs61211515 (T/TT) with C-peptide

	$INFO/R^2$	MAF	BETA*	SE	P	Het I^2	Het P
Stimulated C-peptide							
DCCT Primary Cohort	0.95	0.09	-0.12	0.12	0.346		
DCCT Secondary Cohort, Duration 1-5 Years	0.95	0.09	0.18	0.25	0.460		
DCCT Secondary Cohort, Duration 5-15 Years	0.95	0.09	-0.22	0.22	0.300		
Meta-GWAS			0.09	0.09	0.349	0	0.443
Fasting C-peptide							
DCCT	0.94	0.08	-0.16	0.08	0.062		
CACTI	0.97	0.10	-0.22	0.48	0.642		
EDC	0.96	0.12	-0.77	1.14	0.499		
Meta-GWAS					0.053	0	0.828
Random C-peptide							
WESDR	0.94	0.08	-0.10	0.54	0.848		
Medalist	0.96	0.10	-0.10	0.06	0.128		
Meta-analysis					0.192	0	0.419
Stimulated/Fasting/Random C-peptide Meta-GWAS					0.066	0	0.905

$INFO/R^2$: Quality of imputation

* C-peptide is measured with different assays in pmol/mL in DCCT, Medalist and WESDR; and in pmol/L in CACTI and EDC. It is also natural log transformed.

ESM Table 9: Association of rs61211515 genotype categories with stimulated C-peptide at DCCT eligibility compared to reference (having 2 copies of reference allele)

Genotype	Frequency (%)	BETA	SE	P	LS means (95% CI)*
Del_Del	30 (2.30)	-0.83	0.27	2.07E-3	-2.94 (-3.22, -2.67)
Del_Ref	327 (25.10)	-0.39	0.09	7.10E-6	-2.73 (-2.81, -2.65)
In_Del	31 (2.38)	-0.85	0.26	9.71E-4	-2.99 (-3.26, -2.72)
In_In	7 (0.54)	-0.21	0.48	6.77E-1	-2.67 (-3.24, -2.11)
In_Ref	172 (13.20)	-0.17	0.11	1.13E-1	-2.63 (-2.75, -2.52)
Ref_Ref	736 (56.49)	-	-	-	-2.55 (-2.60, -2.49)

LS: Least square, Del: T deletion, In: T insertion, Ref: Reference
Results are based on Tobit models of stimulated C-peptide at DCCT eligibility and extracted genotypes adjusting for sex, age at diagnosis, T1D duration and cohort (primary vs. secondary). Least square means are calculated based on linear regression.

ESM Table 10: LD between the top SNPs in the MHC region from different meta-GWAS and the SNP tagging *HLA-A*24*

	**rs9260151 Chr6:29911030**	**rs1264813 Chr6:29939900**	**rs61211515 Chr6:30100975**	**rs3135002 Chr6:32668439**
**rs9260151 Chr6:29911030**	-	0.02	0.01	0.00
**rs1264813 Chr6:29939900**	1	-	0.38	0.00
**rs61211515 Chr6:30100975**	0.47	0.85	-	0.00
**rs3135002 Chr6:32668439**	0.03	0.07	0.00	-

The values above and below the diagonal are r^2 and D’, respectively. The values are based on EUR population of the 1000 Genomes Project (phase 3).

ESM Table 11: Association of the top SNPs in the MHC region with stimulated/fasting C-peptide at DCCT eligibility when all three included in the same model

			Stimulated C-peptide		Fasting C-peptide	
	**BP**	**MAF**	**B (SE)**	**P**	**B (SE)**	**P**
**rs9260151 (C>T)**	29,911,030	0.09	0.30 (0.09)	7.65E-4	0.16 (0.08)	4.70E-2
**rs61211515 (T/-)**	30,100,975	0.16	-0.38 (0.07)	1.96E-7	-0.23 (0.07)	6.26E-4
**rs3135002 (C>A)**	32,668,439	0.03	0.35 (0.15)	1.97E-2	0.36 (0.13)	7.83E-3

Alleles are non-effect allele>effect allele.
Associations were tested using QLIM procedure in SAS version 9.4 (SAS, Cary, NC), and are based on extracted genotypes from dosage data according to the best guess using GTOOL, version 0.7.5 (http://www.well.ox.ac.uk/~cfreeman/software/gwas/gtool.html) adjusting for sex, age at diagnosis, T1D duration and cohort (primary vs. secondary).

ESM Table 12: Number of imputed variants from MHC imputation in different studies

	**DCCT**	**CACTI**	**EDC**	**Medalist**	**WESDR**
**Classical HLA Alleles**	424	424	424	424	424
**Polymorphic**	279	233	192	274	217
**R^2 > 0.5**	252	116	115	255	120
**Amino Acid Changes**	1276	1276	1276	1276	1276
**Polymorphic**	1136	1045	1093	1141	1044
**R^2 > 0.5**	1106	812	988	1128	804
**SNPs**	7261	7261	7261	7261	7261
**Polymorphic**	7144	6271	6236	7217	6360
**R^2 > 0.5**	7066	5344	5280	7211	5181
**Total**	8961	8961	8961	8961	8961

R^2: Imputation quality

ESM Table 13: Association of rs1264813, rs689, rs151234, rs12971201 and rs193778 with fasting/random C-peptide in each GWAS and in the meta-analysis

	R^2	MAF	Beta	SE	P	Het I^2	Het P
**rs1264813**							
Fasting C-peptide							
DCCT	1.00	0.11	-0.14	0.07	4.99E-2		
CACTI	1.00	0.13	-0.17	0.43	0.69		
EDC	1.00	0.15	0.02	0.90	0.98		
Meta-analysis					0.073	0.0	0.68
Random C-peptide							
Medalist	1.00	0.13	-0.01	0.05	0.79		
WESDR	1.00	0.14	-0.71	0.48	0.14		
Meta-analysis					0.26	0.0	0.32
Stimulated/Fasting/Random Meta-analysis					4.90E-4	35.3	0.16
**rs689**							
Fasting C-peptide							
DCCT	0.86	0.16	-0.10	0.07	0.14		
CACTI	0.84	0.18	-0.49	0.39	0.22		
EDC	0.83	0.14	-1.96	0.79	0.013		
Meta-analysis					0.011	44.0	0.17
Random C-peptide							
Medalist	0.85	0.15	-0.02	0.05	0.75		
WESDR	0.86	0.17	-0.48	0.39	0.21		
Meta-analysis					0.31	0.0	0.44
Stimulated/Fasting/Random Meta-analysis					7.84E-4	24.1	0.25
**rs151234***							
Random C-peptide							
WESDR	0.55	0.18	0.25	0.47	0.60		
**rs12971201**							
Fasting C-peptide							
DCCT	1.00	0.38	-0.07	0.05	0.12		
CACTI	1.00	0.35	0.09	0.30	0.76		
EDC	1.00	0.36	1.37	0.69	0.047		
Meta-analysis					0.57	67.7	0.045
Random C-peptide							
Medalist	1.00	0.37	-0.06	0.04	0.14		
WESDR	1.00	0.37	0.42	0.29	0.14		
Meta-analysis					0.039	0.0	0.83
Stimulated/Fasting/Random Meta-analysis					0.70	64.8	9.13E-3
**rs193778**							
Fasting C-peptide							
DCCT	1.00	0.26	-0.06	0.05	0.22		
CACTI	0.99	0.29	-0.34	0.32	0.28		
EDC	1.00	0.22	-1.50	0.87	0.08		
Meta-analysis					0.043	0.0	0.46
Random C-peptide							
Medalist	1.00	0.26	-0.07	0.04	0.101		
WESDR	1.00	0.25	-0.08	0.32	0.80		
Meta-analysis					0.15	0.0	0.40
Stimulated/Fasting/Random Meta-analysis					6.47E-3	0.0	0.44

R^2: Quality of imputation
* rs151234 had poor imputation quality in CACTI, WESDR and EDC.

ESM Table 14: Association of *HLA-A*24* and *HLA-A*24:02* with C-peptide

Study	R^2	Freq	HWE P	Beta*	SE	P
*HLA-A*24*						
Stimulated						
DCCT	1.00	0.10	0.26	-0.39	0.09	1.50E-5
Further adjusted for rs61211515	-	-	-	-0.18	0.11	0.09
Fasting						
DCCT	-	-	-	-0.21	0.08	0.01
Random						
Medalist	1.00	0.12	1	-0.11	0.06	0.061
*HLA-A*24:02*						
Stimulated						
DCCT	0.95	0.10	0.26	-0.41	0.10	1.57E-5
Further adjusted for rs61211515	-	-	-	-0.18	0.11	0.10
Fasting						
DCCT	-	-	-	-0.21	0.09	0.01
Random						
Medalist	1.00	0.12	1	-0.11	0.06	0.062

R^2: Quality of imputation
Of the DCCT subjects with *HLA-A*24*, all but one (who was heterozygous for *HLA-A*24:07*) had *HLA-A*24:02*. Both *HLA-A*24* and *HLA-A*24:02* had poor imputation quality in CACTI, EDC and WESDR.
* C-peptide is measured with different assays in pmol/mL in DCCT, Medalist and WESDR; and in pmol/L in CACTI and EDC. It is also natural log transformed.

ESM Table 15: Association of DR3/DR4 categories with stimulated C-peptide at DCCT eligibility

		Primary			Secondary Duration <5 yrs			Secondary Duration >5 yrs			Meta-analysis				
	**N**	**BETA**	**SE**	**P**	**BETA**	**SE**	**P**	**BETA**	**SE**	**P**	**BETA**	**SE**	**P**	**Het I^2**	**Het P**
DR3/X	225	0.02	0.14	0.901	0.62	0.34	0.067	-0.16	0.29	0.585	0.06	0.12	0.596	-	
DR3/DR3	114	0.20	0.18	0.271	0.35	0.39	0.377	-0.29	0.36	0.416	0.14	0.15	0.353	-	-
DR3/DR4	340	0.27	0.14	0.051	0.73	0.31	0.019	-0.09	0.25	0.715	0.26	0.11	0.024	53.10	0.12
X/DR4	323	0.08	0.13	0.546	0.67	0.35	0.055	0.18	0.25	0.474	0.16	0.11	0.152	-	-
DR4/DR4	89	-0.15	0.20	0.446	0.28	0.45	0.534	-0.01	0.37	0.985	-0.07	0.16	0.689	-	-
X/X	212	Ref	-	-	Ref	-	-	Ref	-	-	Ref	-	-	-	-

Het: Heterozygosity
DR3/DR4 categories were defined based on rs2187668 and rs7454108 [10] both genotyped in DCCT.

ESM Table 16: Association of 4 identified variants for C-peptide with T1D [12, 13]

		Affymetrix Platform 1,390 cases & 4,830 Controls				Illumina Platform 3,983 Cases and 3,999 Controls				Meta-analysis 5,913 Cases & 8,828 Controls			
**SNP**	**Alleles**	**INFO**	**OR**	**SE**	**P**	**INFO**	**OR**	**SE**	**P**	**BETA***	**SE†**	**OR**	**P**
rs9260151	C>T	0.91	0.74	0.07	2.63E-06	0.92	0.69	0.05	2.08E-12	-0.35	0.04	0.70	6.43E-17
rs61211515	T/-	0.79	0.98	0.05	0.653	0.81	1.01	0.05	0.829	-0.00	0.04	1.00	0.909
rs3135002	A>C	0.81	27.39	0.20	4.69E-216	0.88	13.57	0.10	9.00E-300	2.75	0.09	15.58	4.96E-209
rs559047	T>A	0.96	0.97	0.05	0.542	0.96	0.96	0.04	0.256	-0.04	0.03	0.96	0.202

Alleles are non-effect allele>effect allele.
* Log-odds ratio
† Standard error of the log-odds ratio

SUPPLEMENTARY Figures

ESM Figure 1: Distribution of stimulated C-peptide at DCCT eligibility

A: Primary Cohort

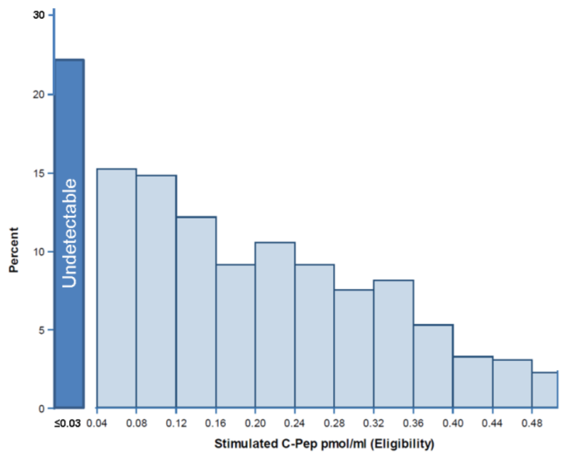

B: Secondary Cohort, Duration 1-5 Years

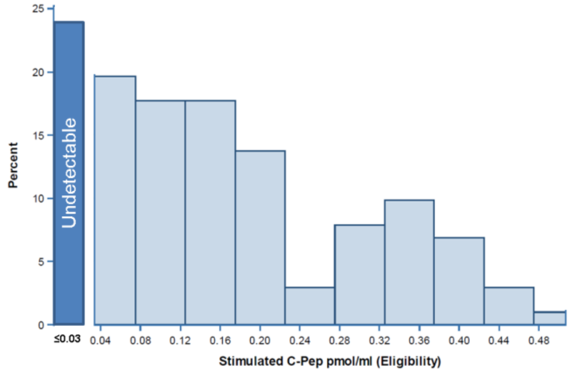

B: Secondary Cohort, Duration 5-15 Years

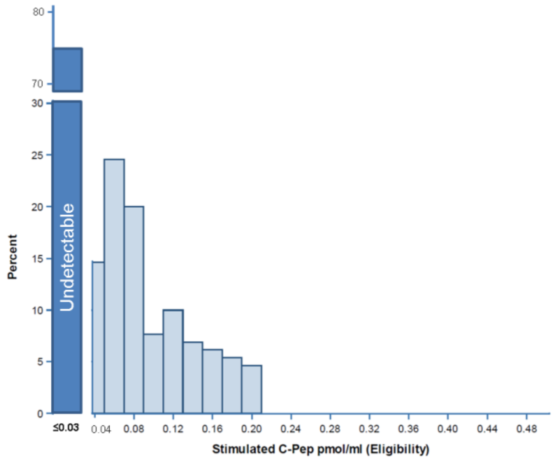

D: All

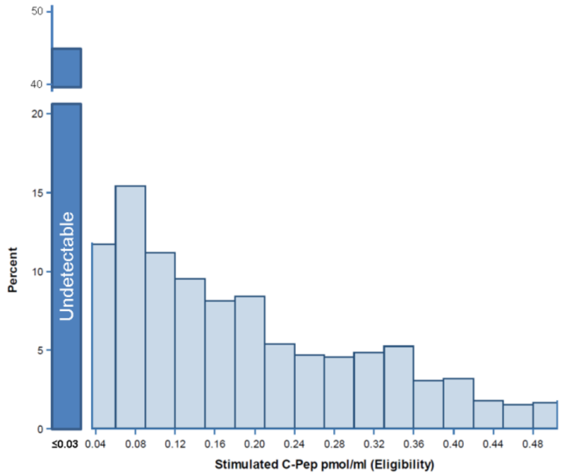

ESM Figure 2: Distribution of fasting/random C-peptide in different cohorts

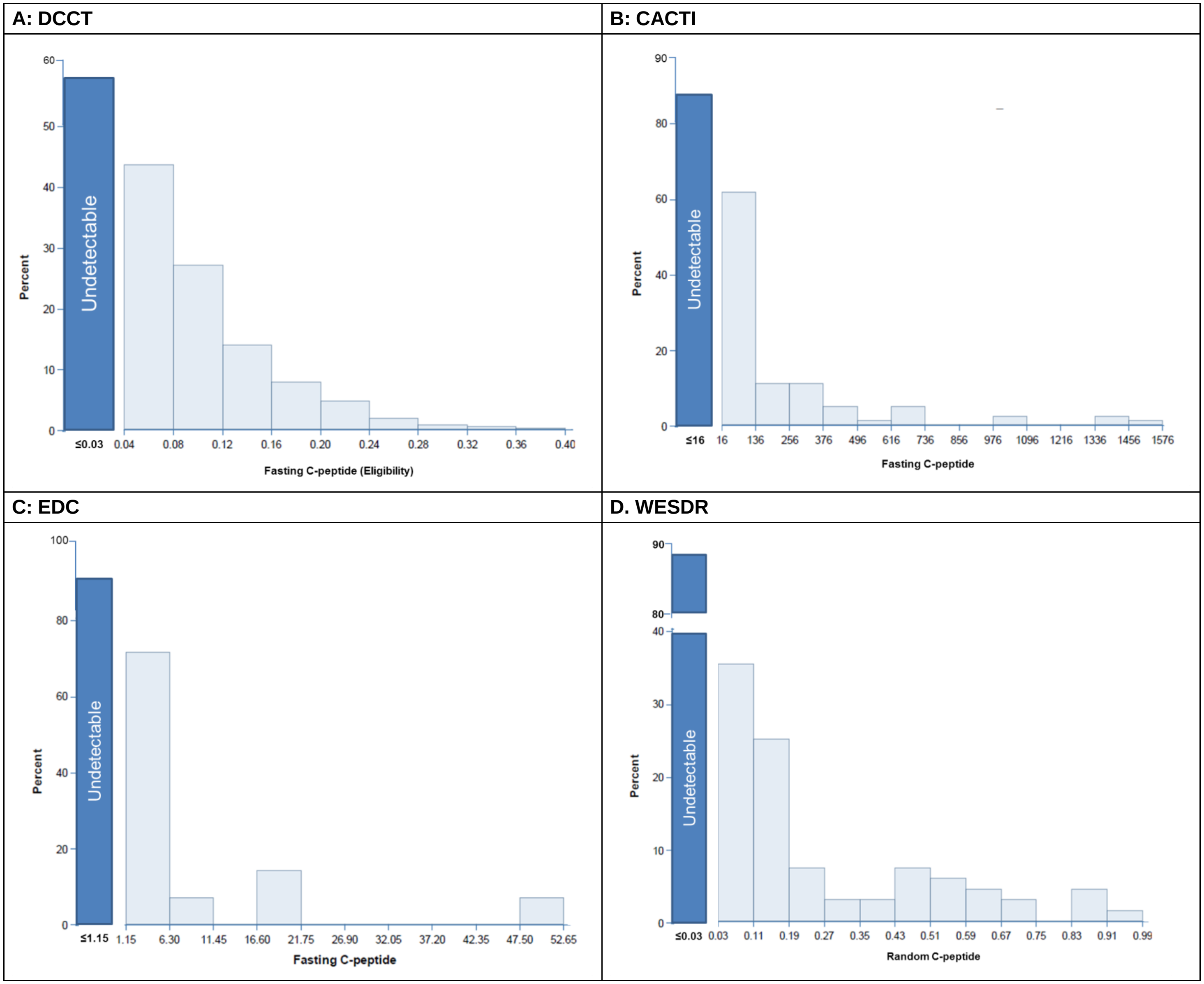

ESM Figure 3: The Manhattan plot of stimulated/fasting/random (A) stimulated (B), fasting (C), random (D) and C-peptide meta-GWAS

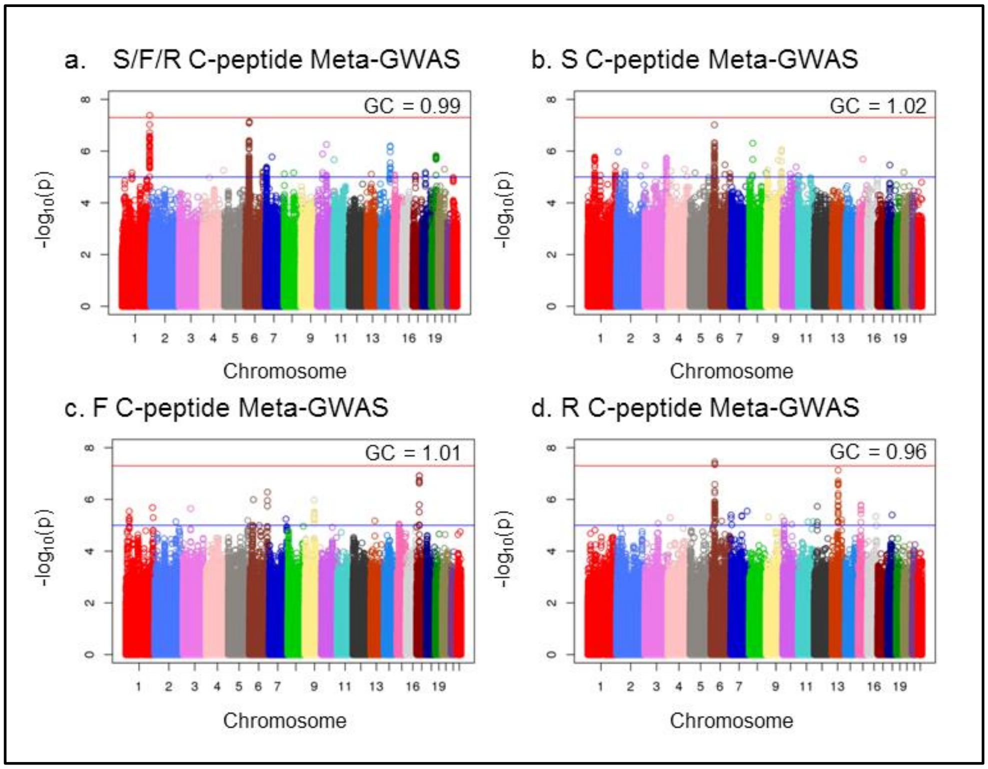

The Manhattan plots for stimulated/fasting/random (S/F/R), stimulated (S), fasting (F) and random (R) C-peptide meta-GWAS show -log_{10}(p-values) (y-axis) of SNPs plotted against their chromosomal positions (x-axis). The red horizontal line represents the genome-wide significant threshold ($p = 5x10^{-8}$) and the blue line represents suggestive association threshold ($p = 1x10^{-5}$). a: Included stimulated C-peptide results from DCCT Primary Cohort, Secondary Cohort with duration 1-5 years, Secondary Cohort with diabetes duration 5-15 years; fasting C-peptide results from CACTI and EDC; and random C-peptide results from Medalist and WESDR. b: Included DCCT Primary Cohort, Secondary Cohort with duration 1-5 years and Secondary Cohort with duration 5-15 years c: Included DCCT, CACTI and EDC d: Included Medalist and WESDR

ESM Figure 4: The QQ-plot of stimulated (A), fasting (B), random (C) and stimulated/fasting/random (D) C-peptide meta-GWAS

A. Stimulated C-peptide

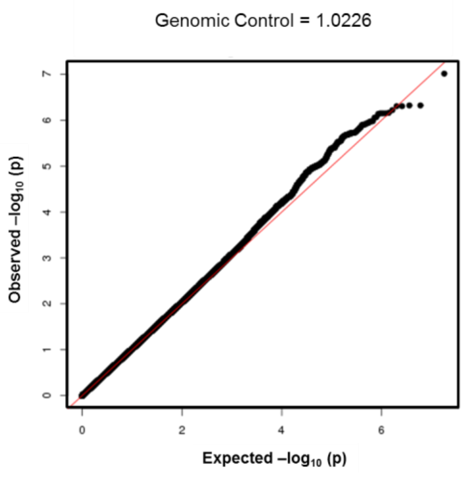

B. Fasting C-peptide

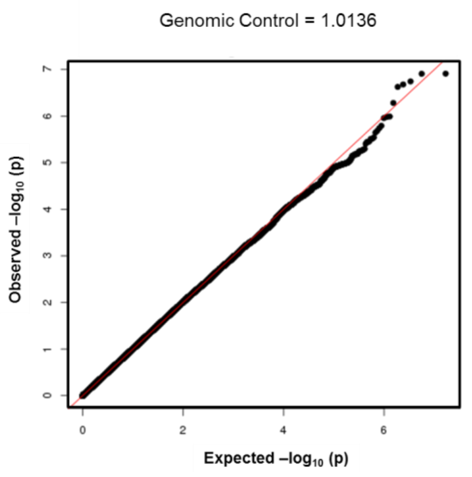

C. Random C-peptide

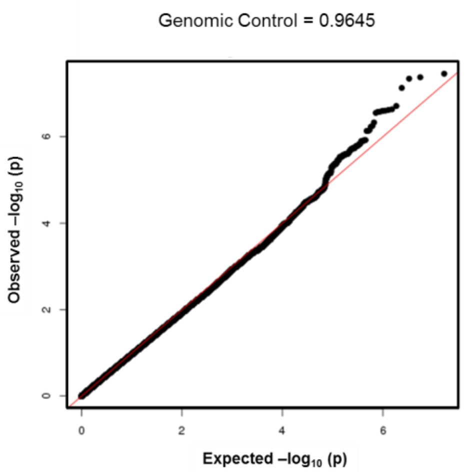

D. Stimulated/Fasting/Random C-peptide

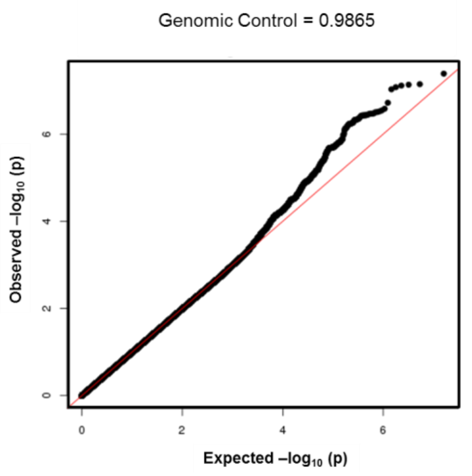

The quantiles of observed versus expected -log_{10}(p-values) regarding SNP (Chr1-22 genotyped and imputed SNPs with minor allele frequency >0.01 and high imputation quality (INFO >0.80 or R^2 >0.5)) associations with Ln(C-peptide) were plotted. A: Included DCCT Primary Cohort, Secondary Cohort with duration 1-5 years and Secondary Cohort with duration 5-15 years B: Included DCCT, CACTI and EDC C: Included Medalist and WESDR D: Included stimulated C-peptide results from DCCT Primary Cohort, Secondary Cohort with duration 1-5 years and Secondary Cohort with duration 5-15 years; fasting C-peptide results from CACTI and EDC; and random C-peptide results from Medalist and WESDR

REFERENCES

[1] Paterson AD, Waggott D, Boright AP, et al. (2010) A genome-wide association study identifies a novel major locus for glycemic control in type 1 diabetes, as measured by both A1C and glucose. Diabetes 59: 539-549

[2] Delaneau O, Marchini J, Zagury JF (2011) A linear complexity phasing method for thousands of genomes. Nature methods 9: 179-181

[3] Delaneau O, Zagury JF, Marchini J (2013) Improved whole-chromosome phasing for disease and population genetic studies. Nature methods 10: 5-6

[4] Auton A, Brooks LD, Durbin RM, et al. (2015) A global reference for human genetic variation. Nature 526: 68-74

[5] Howie BN, Donnelly P, Marchini J (2009) A flexible and accurate genotype imputation method for the next generation of genome-wide association studies. PLoS genetics 5: e1000529

[6] Fuchsberger C, Abecasis GR, Hinds DA (2015) minimac2: faster genotype imputation. Bioinformatics (Oxford, England) 31: 782-784

[7] Howie B, Fuchsberger C, Stephens M, Marchini J, Abecasis GR (2012) Fast and accurate genotype imputation in genome-wide association studies through pre-phasing. Nature genetics 44: 955-959

[8] Oram RA, Patel K, Hill A, et al. (2016) A Type 1 Diabetes Genetic Risk Score Can Aid Discrimination Between Type 1 and Type 2 Diabetes in Young Adults. Diabetes care 39: 337-344

[9] Onengut-Gumuscu S, Chen WM, Burren O, et al. (2015) Fine mapping of type 1 diabetes susceptibility loci and evidence for colocalization of causal variants with lymphoid gene enhancers 47: 381-386

[10] Winkler C, Krumsiek J, Buettner F, et al. (2014) Feature ranking of type 1 diabetes susceptibility genes improves prediction of type 1 diabetes. Diabetologia 57: 2521-2529

[11] Barker JM, Triolo TM, Aly TA, et al. (2008) Two single nucleotide polymorphisms identify the highest-risk diabetes HLA genotype: potential for rapid screening. Diabetes 57: 3152-3155

[12] Censin JC, Nowak C, Cooper N, Bergsten P, Todd JA, Fall T (2017) Childhood adiposity and risk of type 1 diabetes: A Mendelian randomization study. PLoS medicine 14: e1002362

[13] Censin JC, Nowak C, Cooper N, Bergsten P, Todd JA, Fall T (2017) Data from: Childhood adiposity and type 1 diabetes: a Mendelian randomization study In, Dryad Digital Repository
